# Heat Index: An Alternative Indicator for Measuring the Impacts of Meteorological Factors on Diarrhoea in the Climate Change Era: A Time Series Study in Dhaka, Bangladesh

**DOI:** 10.3390/ijerph21111481

**Published:** 2024-11-07

**Authors:** Farhana Haque, Fiona C. Lampe, Shakoor Hajat, Katerina Stavrianaki, S. M. Tafsir Hasan, A. S. G. Faruque, Tahmeed Ahmed, Shamim Jubayer, Ilan Kelman

**Affiliations:** 1 Institute for Global Health (IGH), University College London (UCL), London WC1N 1EH, UK; f.lampe@ucl.ac.uk; 2 UK Public Health Rapid Support Team (UK PHRST), Department of Infectious Disease Epidemiology, London School of Hygiene and Tropical Medicine (LSHTM), London WC1E 7HT, UK; 3 Department of Public Health, Environments and Society, London School of Hygiene and Tropical Medicine (LSHTM), London WC1H 9SH, UK; shakoor.hajat@lshtm.ac.uk; 4 Department of Statistical Science, Department of Risk and Disaster Reduction, University College London (UCL), London WC1E 6BT, UK; k.stavrianaki@ucl.ac.uk; 5 Nutrition Research Division (NRD), International Centre for Diarrhoeal Disease Research, Bangladesh (icddr,b), Dhaka 1212, Bangladesh; tafsir.hasan@icddrb.org (S.M.T.H.); gfaruque@icddrb.org (A.S.G.F.); tahmeed@icddrb.org (T.A.); 6 Department of Epidemiology and Research, National Heart Foundation Hospital and Research Institute, Dhaka 1216, Bangladesh; jubayers@nhf.org.bd; 7 Institute for Global Health (IGH) and Department of Risk and Disaster Reduction, University College London (UCL), London WC1E 6BT, UK; i.kelman@ucl.ac.uk; 8 Campus Kristiansand, University of Agder, 4630 Kristiansand, Norway

**Keywords:** heat index, diarrhoeal disease, HI, diarrhoea, Bangladesh

## Abstract

Heat index (HI) is a biometeorological indicator that combines temperature and relative humidity. This study aimed to investigate the relationship between the Heat Index and daily counts of diarrhoea hospitalisation in Dhaka, Bangladesh. Data on daily diarrhoea hospitalisations and meteorological variables from 1981 to 2010 were collected. We categorised the Heat Index of >94.3 °F (>34.6 °C), >100.7 °F (>38.2 °C) and >105 °F (>40.6 °C) as high, very high and extremely high Heat Index, respectively. We applied a time series adjusted generalised linear model (GLM) with negative binomial distribution to investigate the effects of the Heat Index and extreme Heat Index on hospitalisations for diarrhoea. Effects were assessed for all ages, children under 5 years old and by gender. A unit higher HI and high, very high and extremely high HI were associated with 0.8%, 8%, 7% and 9% increase in diarrhoea hospitalisations in all ages, respectively. The effects varied slightly by gender and were most pronounced in children under 5 years old with a rise of 1°F in high, very high and extremely high HI associated with a 14.1% (95% CI: 11.3–17.0%), 18.3% (95% CI: 13.4–23.5%) and 18.1% (95% CI: 8.4–28.6%) increase of diarrhoea, respectively. This suggests that the Heat Index may serve as an alternative indicator for measuring the combined effects of temperature and humidity on diarrhoea.

## 1. Introduction

Air temperature alone is seldom regarded as the best indicator for measuring the amount of heat stress on humans caused by excessive levels of air temperature [1,2,3]. Relative humidity, defined as the amount of moisture in the air, compared to what the air can hold at that temperature, may affect heat stress [2,4]. Since the conditions of heat exchange between the human body and the surrounding thermal environment are quite complex, thermal indices, mostly involving two parameters, have been developed. Multiple studies have evaluated the effectiveness and ease of use of these indices to better capture the risks associated with changes in the thermal environment [5,6,7]. For tropical countries, such indices frequently combine the air temperature and different measures of humidity [1,5]. One index for thermal stress assessment that is widely used by various national and local weather services in the US is known as the Heat Index (HI). The HI is a biometeorological index that measures heat stress in the human body by combining air temperature with relative humidity [8]. While no indicator is perfect, heat stress indicators that consider multiple parameters, including temperature and humidity, have been shown to consistently perform better than simpler metrics [5,6,7].

With global climate change, significant upward trends in the HI have been reported in many humid regions across the globe, including the United States, Southeast Asia, India, Europe, and Northern Australia [9,10,11,12,13,14,15]. This index can, therefore, be an alternative indicator of temperature and could better reflect the exposure-response relationship between temperature and health in the context of climate change. Adverse health consequences have been associated with periods of very high HI in many countries, including the US, Vietnam, Taiwan, Bangladesh, and India [9,16,17,18,19,20,21,22].

Diarrhoea occurs when more than three loose, watery stools are passed within 24 h. Although the exact prevalence and incidence of diarrhoea are not available, diarrhoea is endemic and a major cause of morbidity and mortality in Bangladesh [23]. With notable improvements in diarrhoea care, Bangladesh has achieved significant success in reducing diarrhoea mortality in recent years [24,25]. However, diarrhoea outbreaks and hyper endemicity continue to plague the nation despite notable improvements in socioeconomic conditions as well as in water and sanitation infrastructure [26]. The impact of environmental temperature on diarrhoeal diseases has been studied extensively [27,28,29,30]. Prior studies have also explored the effects of humidity on diarrhoea [30]. However, the combined impact of various meteorological parameters, such as HI, on diarrhoea remains relatively less explored [29,30]. While the potential impact of HI on diarrhoea is currently underexplored, it is well established that both temperature and relative humidity can directly affect the replication of diarrhoeal pathogens, including bacteria and protozoa, as well as enhance the survival of enteroviruses [31,32,33,34]. Because heat indices may be able to capture better risk levels associated with the thermal environment [6], the HI may serve as an important exposure for determining the combined impact of environmental temperature and humidity on diarrhoea in the context of rising heat stress due to climate change [35].

Given that a recent systematic review found no previous studies assessing the effect of HI on diarrhoea hospitalisations [36], this study aimed to identify the relationship between HI and hospitalisations for diarrhoea, examine the impacts of extreme HI, and elucidate the modifying effects of age, gender, and season in Dhaka Bangladesh. In addition, the performance of HI as a heat stress indicator was compared to simpler metrics, including mean temperature. Although it is acknowledged that correlations uncovered do not necessarily imply direct causation, such indicators may support an understanding of the effects of climate parameters on diarrhoeal disease morbidity, thereby aiding further research to elicit linkages between climate change and gastrointestinal health.

## 2. Materials and Methods

### 2.1. Study Setting

Dhaka, the capital of Bangladesh, is located at 23°42′ N 90°22′ E, on the eastern banks of the Buriganga River. The low-lying coastal city surrounded by five major rivers and 50 water channels is at risk of flooding from rising sea levels [37]. Several dykes and embankments, and the interim boundaries created to protect Dhaka from flooding, together with inadequate drainage and water pumping systems, frequently result in waterlogging of the protected city area during the rainy monsoon season [38,39]. With over 20 million residents, the rapidly expanding, resource-constrained megacity faces a number of challenges, including inadequate water, sanitation and hygiene infrastructure [39,40]. Given these challenges, the impacts of meteorological parameters on diarrhoea could be particularly concerning for Dhaka, where diarrhoea is already highly prevalent [23].

### 2.2. Diarrhoea Data

Daily data on the total number of patients of all ages seeking care for diarrhoea (defined as the passage of three or more loose stools per 24 h due to any cause) by age group and gender between 1 January 1981 and 31 December 2010 were collected from the Dhaka Hospital of the International Centre for Diarrhoeal Diseases Research, Bangladesh (icddr,b) on 7 October 2020. Since reliable records of the total number of patients admitted per day and the illness onset dates were not available for the study period, we collected data from icddr,b’s Diarrhoeal Disease Surveillance System (DDSS) to estimate the total number of diarrhoea patients per day. The DDSS is a robust surveillance system that was established in 1979 to collect information from a systematic subsample of patients attending the Dhaka Hospital. During 1979 and 1995, every 25th patient (4% of the sample) and during 1996 and 2010, every 50th patient (2% of the sample) was enrolled on the DDSS. Surveillance patients were seen by the regular hospital staff, and emergency cases were treated on a priority basis. All patients were first treated in an outpatient area; those requiring further care were admitted to an intravenous treatment centre or a hospital ward. After initial examination and care by a nurse or doctor, a trained health assistant sought verbal informed consent from the surveillance patient or an adult guardian prior to the interview. No personal identifying information was accessed for this study. The well-known diarrhoeal disease hospital in Dhaka served a population of approximately 3.5 million in 1981, 6.6 million in 1990 and 14.6 million in 2010, predominantly from urban Dhaka [36]. Additional information on health data is provided in the Supporting Information (Supplementary File S1).

### 2.3. Meteorological Data

Daily data on the mean temperature (°C), relative humidity (%), atmospheric pressure (hPa) and cumulative rainfall (mm) for Dhaka were obtained from the Bangladesh Meteorological Department (BMD) for the study period. The BMD recorded 3-hourly temperature data from three validated weather stations within Dhaka (https://dataportal.bmd.gov.bd/) (accessed on 7 October 2020) [41].

### 2.4. Calculating the Heat Index (HI)

For this study, the HI was calculated using the formula described in a 1990 National Weather Service (NWS) Technical Attachment (SR 90-23) [42].

Heat Index (HI) = − 42.379 + (2.04901523 × Tf) + (10.14333127 × RH) − (0.22475541 × Tf × RH) − (6.83783 × 10^−3^ × Tf^2^) − (5.481717 × 10^−2^ × RH^2^) + (1.22874 × 10^−3^) Tf ^2^(RH) + (8.5282 × 10−4 × Tf × RH^2^) − (1.99 × 10^−6^) Tf^2^ RH^2^

where:

Tf is the average air temperature in degrees Fahrenheit

RH is the relative humidity using whole numbers

### 2.5. Statistical Analysis

A descriptive analysis was performed to understand the daily time series characteristics of diarrhoea and HI over the study period. Each data series was checked for stationarity, autocorrelation, long-term trends, seasonality, possible outliers, normality, homoscedasticity and volatility using established methods [43,44,45,46,47]. The HI data series was pre-whitened using autoregressive integrated moving average (ARIMA) models to achieve stationarity before checking the cross-correlation between diarrhoea hospitalisations and HI.

A time series adjusted generalised linear model (GLM) with negative binomial regression distribution was used to assess the association between acute effects of HI and extreme HI on diarrhoea hospitalisations [46]. The negative binomial distribution was used to allow for the overdispersion/extra variation detected in the response variable [48]. Since past studies have identified the atmospheric pressure, day of the week, long-term time and seasonal trends, and rainfall as potential confounders of the relationship between HI and human health, the model was adjusted for these potential confounding factors [49]. To examine the effect of season, an indicator variable for the season was incorporated into the model. Additional details on potential confounders/covariates and effect modifiers are provided in the Supplementary Materials (Supplementary File S2). The months of November to February were defined as the winter, March to June as the summer and July to October as the rainy monsoon season, reflecting the seasonality of Bangladesh [50]. Given that most studies investigating the lagged effects of temperature considered the lagged effects for 4 weeks [27], while a study reported weak nonlinear effects of the relative humidity on diarrhoea for 1 to 6 weeks [51], we initially investigated individual and distributed lag effects for 28 days. As a statistically significant relationship between diarrhoea and HI was found at lag zero only, and the lagged model performed poorly in terms of model diagnostics and analysis of residuals, lag effects were not considered in the model. A series of flexible spline functions of time were used to flexibly model the underlying trends and seasonal patterns in the health data unrelated to climatic factors [43]. The Bayesian Information Criterion (BIC) and analysis of residuals were used to evaluate the model fit and inform the choice of degrees of freedom for flexible splines. Model residuals were lagged by one day to adjust for significant residual autocorrelation associated with the day before. For all statistical tests, values of *p* < 0.05 were considered statistically significant. However, *p*-values were not adjusted for the number of statistical tests performed. The relative risk of hospitalisation for diarrhoea per unit change in HI was calculated from Equation (1) as incidence rate ratio (IRR).

The model to investigate the effects of HI on diarrhoea took the following form:

Y_t_ ~ Negative Binomial (µ_t_, θ)

log[E(Y_t_)] = β_0_ + β_1_HI_1t_ + β_2_Rain_2t_ + β_3_ATPress_3t_ + γ_1_dow_1t_ + γ_2_Season_2t_ + θ_1_(LS Time, 8 D.F.)_1t_ (1)

where E(Y_t_) denotes the expected daily diarrhoea count at time t, log[E(Y_t_)] is the natural logarithm of the expected count (log link function), β_0_ is the intercept term, HI_1t_ denotes the Heat Index at time t, Rain_t_ and ATPress_t_ denote the cumulative rainfall and daily atmospheric pressure at time t, respectively. To control for long-term trends and seasonality, a flexible linear spline LS with 8 degrees of freedom per year was incorporated into the model. Season is the categorical variable denoting the three seasons, and dow_t_ is the categorical day of the week with a reference day of Friday.

To explore the impacts of extreme HI on diarrhoea, three categories of HI were generated. The heat indices above the 75th, 95th and 99th percentiles during the study period were defined as high HI (>94.3 °F/34.6 °C), very high HI (>100.7°F/38.2 °C) and extremely high HI (>105 °F/40.6 °C), respectively. To examine the effect of extreme HI on diarrhoea, different models were generated using the three categories of extreme HI.

In addition, we used a generalised linear model to study the effects of temperature and relative humidity on diarrhoea hospitalisations. The model is as follows:

log[E(Y_t_)] = β_0_ + β_1_Temp_1t_ + β_2_Hum_2t_ + β_3_Rain_3t_ + β_4_ATPress_4t_ + γ_1_dow_1t_ + γ_2_Season_2t_ + θ_1_(LS Time, 8 D.F.)_1t_ (2)

where E(Y_t_) denotes the expected daily diarrhoea count at time t, log[E(Y_t_)] is the natural logarithm of the expected count (log link function), β_0_ is the intercept term, Temp_1t_ and Hum_2t_ denote mean temperature and relative humidity at time t, respectively, Rain_t_ and ATPress_t_ denote the cumulative rainfall and daily atmospheric pressure at time t, respectively. LS is a flexible linear spline with 8 degrees, Season is the categorical variable representing the three seasons, and dow_t_ is the categorical day of the week with a reference day of Friday.

The estimation of relative risks can change significantly with changing model specifications [49,52]. As a result, the analyses were repeated using linear splines of 1–14 degrees of freedom per year and by fitting a natural cubic spline with 3–7 D.F. to control for the long-term trend and seasonality instead of linear splines. In addition, sensitivity analyses were conducted, removing atmospheric pressure from the covariates and including interaction terms in the temperature humidity models. We reran the analyses using the number of patients enrolled into the icddr,b’s DDSS as the outcome instead of the total estimated diarrhoea hospitalisations per day. We used Stata/SE 18.0 (StataCorp LLC, Texas, USA) for the data analyses in this study.

### 2.6. Ethics Approval

This study was granted approval by the Research Review Committee (RRC) and Ethical Review Committee (ERC) of the International Centre for Diarrhoeal Diseases Research, Bangladesh (icddr,b) (Protocol number: PR-19097). This study used secondary data and did not involve primary data collection from human participants. This study was also approved by the University College London’s Research Ethics Committee (UCL REC).

## 3. Results

**Figure 1:**
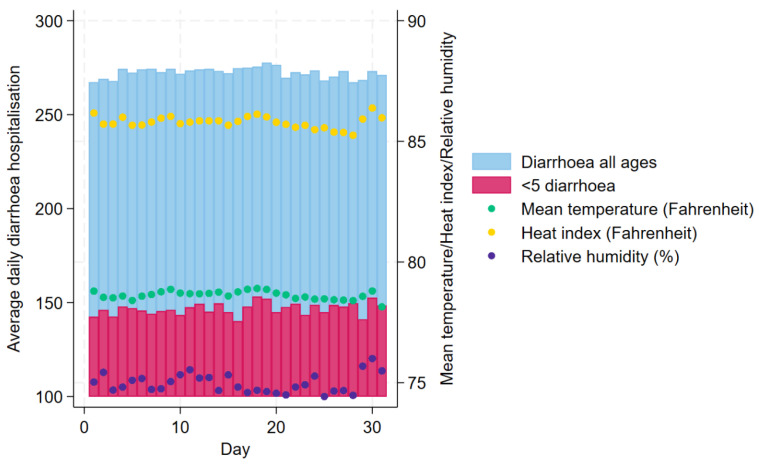
Daily distribution of diarrhoea hospitalisations in all ages and children under 5 years old and Heat Index, ambient temperature, and relative humidity averaged between 1981 and 2010 in Dhaka, Bangladesh.

**Figure 2:**
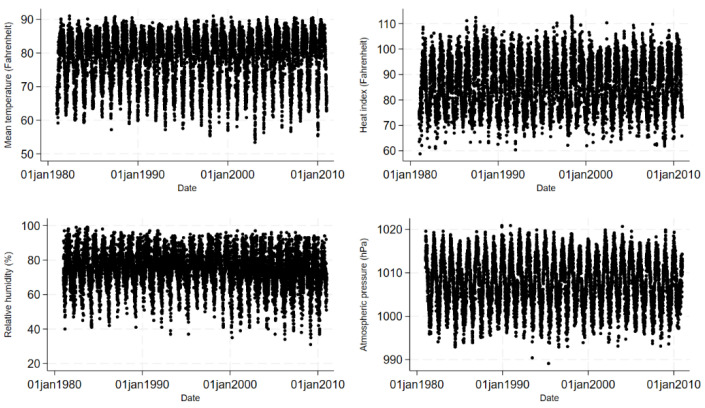
Daily mean temperature (Fahrenheit), daily relative humidity (%), daily atmospheric pressure (hPa) and Heat Index (Fahrenheit) from 1981 to 2010 in Dhaka, Bangladesh.

**Figure 3:**
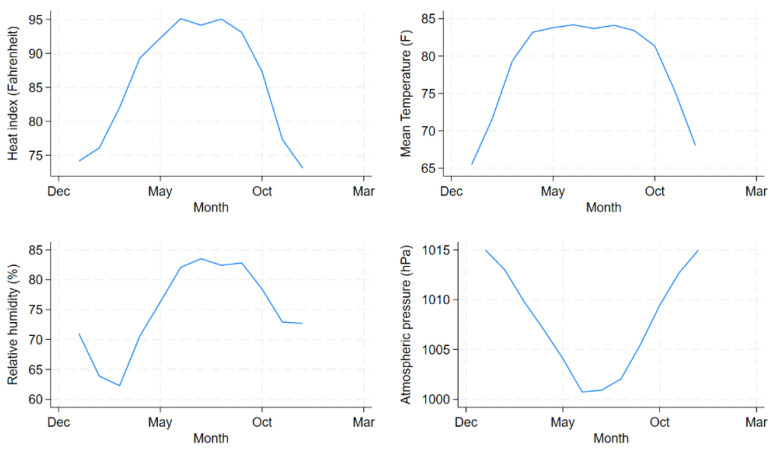
Monthly distributions of the daily mean temperature (F), daily relative humidity (%), daily atmospheric pressure (hPa) and Heat Index (Fahrenheit) from 1981 to 2010 in Dhaka, Bangladesh.

Approximately 2,983,850 diarrhoea patients sought care from the icddr,b Dhaka Hospital during the 30-year study period. Figure 1 shows the daily distributions of diarrhoea hospitalisations for all ages and children under 5 years old and mean temperature (°F), HI (°F) and relative humidity (%) in Dhaka, averaged across the years 1981 and 2010. Figure 2 and Figure 3 show the daily and monthly distributions of meteorological factors in Dhaka, Bangladesh, respectively, revealing distinct seasonal patterns. HI remained high from April to September, peaking during June and August, and started to decline since October. The figure suggests that HI was lower from December to February, which corresponds roughly to the winter season in Bangladesh. Table 1 shows the summary statistics for HI, mean temperature, relative humidity, atmospheric pressure, and diarrhoea hospitalisations. During the study period, the median HI was 86.2 °F (30.1 °C), the median temperature was 81.1 °F (27.3 °C), and the median relative humidity was 76.0%. The median number of daily diarrhoea admissions in all ages for the period was 250. Only 25 out of 10,957 (0.2%) days during the study period had a relative humidity lower than 40%.

**Figure 4:**
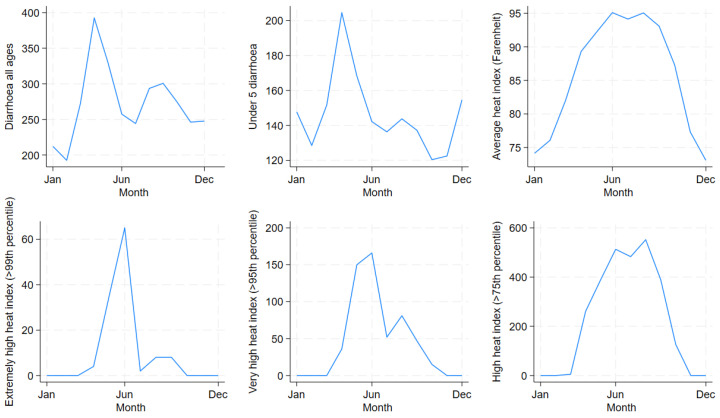
Monthly distribution of diarrhoea hospitalisations in all ages and children under 5 years old in the Dhaka Hospital, Heat Index and extreme Heat Index days in Dhaka, Bangladesh from 1981 to 2010.

Figure 4 shows the temporal distribution of diarrhoea hospitalisations in all ages and children under 5 years old and the extreme HI indicators by month. Diarrhoea hospitalisations in all ages and children under 5 years old peaked in April. Most extreme HI days were concentrated during the summer months. Extremely high (>99th percentile) and very high HI (>95th percentile) peaked in June, whereas high HI (>75th percentile) peaked in September. Table 2 shows a decreasing trend of extreme HIs across decades. However, the residents of Dhaka were still exposed to a considerable number of large HIs during the 30-year study period.

As highlighted in Table 3, a 1 °F rise in HI, high, very high and extremely high HI was associated with a 0.84% (95% CI: 0.76–0.91%; *p* < 0.001), 7.7% (95% CI: 6.7–8.8%; *p* < 0.001), 7.3% (95% CI: 5.5–9.0%; *p* < 0.001) and 8.8% (95% CI: 5.3–12.4%; *p* < 0.001) increase in diarrhoea, respectively. The overall impact of HI varied slightly by gender (1.1% in males vs. 1.0% in females). The effects were greatest in children under 5 years old.

Using Equation (2), 1 °F increase in mean temperature was associated with 1.4% (1.3–1.5), 1.8% (1.4–2.1), 1.1% (0.9–1.3) and 1.0% (0.7–1.3) increase in diarrhoea hospitalisation in all ages, children under 5 years old, males, and females, respectively. The results of the sensitivity analyses changed the results very little (Supplementary File S3). Acceptable model fit was achieved for the models in the various subgroups. Partial autocorrelation plots of the deviance residuals show that for all sub-groups, the Heat Index models had negligible deviance residuals (Supplementary File S3).

## 4. Discussion

This study examined the effects of the HI on diarrhoea in all ages and found a small but significant nonlinear positive relationship between the HI and diarrhoea after controlling for the confounding effects of atmospheric pressure, heavy rainfall, day of the week, long-term time trend and seasonality. A unit (1 °F) increase in the HI was associated with a 0.8–0.9% increase in the risk of hospitalisation for diarrhoea. While no previous study has investigated the effects of the HI on diarrhoeal disease morbidity, a few past studies examining the impact of HI on mortality and cardiovascular diseases have reported a non-linear relationship between the HI and health outcomes [49,53].

In this study, the effects of a relatively large HI were found to be much more pronounced than the average HI. Between 1981 and 2010, the population in Dhaka were exposed to high (>94.3 °F/34.6 °C), very high (>100.7 °F/38.2 °C) and extremely high (>105 °F/40.6 °C) HI for 2718, 547 and 122 days, respectively. High, very high and extremely high HI were associated with approximately 8%, 7% and 9% increase in the risk of diarrhoea in all ages, respectively. The results of this study support the hypothesis that large HI poses a greater risk to human health, including diarrhoea.

With ongoing climate change, the HIs are likely to become more and more variable. As pointed out by several past investigations, the effects of an increased HI on health outcomes, including diarrhoea, are likely to become more severe with global climate change. Although the dose-response relationship between exposure to various levels of HI and diarrhoeal disease morbidity has not been investigated in the past, the US National Weather Service generates different heat alerts according to the level of exposure to HIs and heat illnesses [8]. The result of this study underscores the importance of raising awareness among the patients, caregivers, medical, public health and allied staff of the particularly high risk posed by relatively large HIs on diarrhoeal disease morbidity among the residents of Dhaka. Based on the impact of extreme HI on morbidity, similar heat alerts could be generated not only for heat-related illnesses but also for other morbidity, including diarrhoea that likely impacts people even within indoor settings in addition to outdoors.

In this study, children under 5 years old were observed to experience greater impacts of relatively large HIs compared to all ages. Unit increases in very high and extremely high HIs were associated with an approximately 18% increase in diarrhoea hospitalisations among children under 5 years old. While the exact mechanism by which exposure to large HIs influences the risk of diarrhoea in young children remains largely unknown, the immune system in children is likely to be immature, and children are likely to have low self-care capacity [41,54,55,56,57].

In addition to young children, males were found to be at a slightly higher risk of diarrhoea due to extremely high (14.2% vs. 10.4%) and high HI (10.1% vs. 8.4%) compared to females. However, compared to males, females were found to be at slightly higher risk for very high HI (10.2% vs. 10.8%). A few studies that have investigated the impact of HI on cardiovascular disease mortality have reported a greater vulnerability of females to the effects of HI owing to their pathophysiological responses to heat stress [19,49,58,59]. The inconsistency noted in this study suggests that additional factors, including social and living conditions [60], social behaviour, amount of time spent outdoors vs. indoors, and healthcare-seeking behaviour, likely also modify the effect of HIs on diarrhoea hospitalisations. Future studies focused on identifying vulnerable groups based on the impacts of HIs on specific morbidities could shed further light on the modifying role of gender.

Although forecasting a Heat Index may not be as straightforward as a temperature forecast, the negligible deviance residuals in the Heat Index models in this study suggest that Heat Index may perform similarly to temperature as an indicator to measure the effects of meteorological factors on diarrhoeal diseases in Dhaka, Bangladesh. This finding is consistent with a previous study that examined the utility of various measures of temperature to identify the best predictor of mortality and found no single measure to be superior to the others [5,53]. However, this finding is inconsistent with the finding of a study conducted by Yin and Wang in 2018 in China, where the Heat Index performed better than the mean temperature to measure the effects of meteorological parameters on CVD mortality [6,49]. While the exact mechanism of how temperature and relative humidity together affect diarrhoea occurrence is yet to be elicited, temperature and relative humidity likely affect the host immunity, host susceptibility, pathogen transmission and pathogen survival, thereby indirectly affecting diarrhoeal disease morbidity [30]. On the other hand, meteorological parameters exert a direct effect on CVD mortality by compromising the body’s capacity to lose heat and control the core body temperature via sweat evaporation [61]. As a result, the observed difference in the HI effect could result from the difference in the way temperature and relative humidity affect the causation of infectious diarrhoea as opposed to CVD mortality.

While this study used established statistical techniques to quantify the effects of the HI and extreme HI on 30 years of clinically certified, high-quality diarrhoea hospitalisation data, and further examined the variations of the HI impact by gender and age groups, this ecological regression study has several limitations. Diarrhoea occurrence and hospitalisation are determined not only by meteorological factors, including temperature and relative humidity, but also by other individual and socioeconomic factors, such as nutritional status, educational level, water and sanitation infrastructure, poverty and healthcare-seeking behaviour, which may vary among cities. These additional factors should be taken into consideration when comparing results from different areas. Although unlikely to affect the analysis of trends, the change in surveillance frequency between 1981 to 1995 and 1996 to 2010 may have introduced some bias. In addition, the study period consisted of 25 days of observation with relative humidity below 40%. The HI in this study was derived from the formula proposed by Rothfusz, which is known to work better at a relative humidity equal to or higher than 40% [42]. This may have introduced some bias into the results. Given that only 0.2% of the data points represented such malformation, a relative humidity of less than 40% was unlikely to deviate the results significantly. Furthermore, sensitivity analysis conducted by replacing the 25 data points with the mean relative humidity did not change the result. Huang et al. (2011) adapted the Heat Index formula for Beijing, China, which was not only suitable for the climatic conditions of that country but also applicable to a relative humidity below 40% [49]. Future studies can compare the utility of both formulas for analysing the relationship between HI and health outcomes in the Bangladeshi context. Given that the temperature sensitivity of diarrhoeal diseases can vary according to the enteropathogen causing the diarrhoea [62], studies utilising pathogen-specific data in modelling the heat-index-diarrhoea relationship may help to generate better estimates. In addition, future studies using recent data and representing various climatic and sociodemographic contexts may help to validate the findings from this study. Although this ecological regression analysis cannot provide insight into the mechanisms underlying the relationship between the HI and diarrhoea hospitalisations, the associations detected strongly justify follow-up investigations. Studies using other data sources and study types may further validate our findings, as well as shed light on mediating factors, effect modifiers, biological mechanisms, and relationships between the HI and other diarrhoeal disease outcomes such as incidence and mortality.

## 5. Conclusions

In an era of climate change, the effects of the HI on diarrhoea are likely to become more pronounced, particularly among children under 5 years old. This underscores the importance of conducting research to elucidate the impacts of the HI on diarrhoea as well as to develop targeted health policies and programs to mitigate the risks. Using a comprehensive meteorological index, the Heat Index, to analyse the combined effects of temperature and relative humidity on diarrhoeal diseases, this study advances the knowledge of how to measure morbidity risk using alternative weather indicators.

## Figures and Tables

**Table 1:** Summary statistics of the daily Heat Index (HI), relative humidity, atmospheric pressure and diarrhoea hospitalisations in all ages by decades from 1981 to 2010 in Dhaka, Bangladesh.

Period	Heat Index, HI (°F) Median (Interquartile Range, IQR)	Daily Average Temperature (°F) Median (IQR)	Relative Humidity (%) Median (IQR)	Atmospheric Pressure (hPa) Median (IQR)	Daily Diarrhoea Hospitalisation Median (IQR)
1981–1990	85.6 (18.1)	80.8 (11.2)	77 (13)	1007.8 (9.7)	175 (75)
1991–2000	86.3 (18.1)	81.0 (11.5)	77 (11)	1007.8 (9.5)	300 (125)
2001–2010	86.7 (17.2)	81.5 (11.2)	74 (14)	1008 (9.2)	300 (150)
1981–2010	86.2 (17.7)	81.1 (11.3)	76 (13)	1007.9 (9.5)	250 (125)

**Table 2:** Decadal distribution of categories of extreme Heat Index in Dhaka, Bangladesh (1 January 1981 to 31 December 2010).

Year	Total Number of Days with High HI (Above 75th Percentile: >94.3 °F/34.6 °C)	Total Number of Days with Very High HI (Above 95th Percentile: >100.7 °F/38.2 °C)	Total Number of Days with Extremely High HI (Above 99th Percentile: >105 °F/40.6 °C)
1981–1990	913	218	48
1991–2000	914	202	47
2000–2010	891	127	27
1981–2010	2,718	547	122

**Table 3:** Percentage increase in diarrhoea hospitalisation associated with one unit increase in Heat Index and extreme Heat Index in Dhaka, Bangladesh.

HI Category	Percentage Increase (95% CI)			
	All Ages	Under-5 Children	Males	Females
HI (°F)	**0.8 (0.8–0.9)**	**1.3 (1.1–1.5)**	**1.1 (0.9–1.3)**	**1.0 (0.7–1.3)**
High HI (>98.4 °F)	**6.9 (5.6–8.3)**	**13.4 (9.8–17.0)**	**10.1 (6.7–13.5)**	**8.4 (3.5–13.4)**
Very high HI (>100.7 °F)	**7.3 (5.5–9.0)**	**18.3 (13.4–23.5)**	**9.2 (4.8–13.8)**	**10.0 (3.6–16.9)**
Extremely high HI (>105 °F)	**8.8 (5.3–12.4)**	**18.1 (8.4–28.6)**	**14.2 (5.2–24.0)**	10.4 (1.0–24.7)

Bold values are significant; significance was defined as *p*-value < 0.05.

## Data Availability

The datasets presented in this article are not readily available because, according to the institutional data policy of the icddr,b (International Centre for Diarrhoeal Disease Research, Bangladesh), only a summary of the data can be publicly displayed or can be made publicly accessible. To protect the intellectual property rights of primary data, icddr,b cannot make primary data publicly available. However, upon request, the Institutional Data Access Committee of icddr,b can provide access to primary data to any individual upon reviewing the nature and potential use of the data. Requests to access the datasets should be directed to Ms. Armana Ahmed, Head, Research Administration, icddr,b, Dhaka, Bangladesh, Email: aahmed@icddrb.org, Phone: +88-02-9827001-10 (ext. 3200).

## Supplementary Materials

The following supporting information can be downloaded at https://www.mdpi.com/article/10.3390/ijerph21111481/s1, Supplementary File S1: Description of the health data (References [36,41] are cited in the supplementary material), Supplementary File S2: Conceptual Framework (References [29,30,32,33,34,35,36,43,47] are cited in the supplementary material), Supplementary File S3: Sensitivity analyses and model diagnostics (References [43,44,45,46] are cited in the supplementary materials).
