# Supplementary material for: Heat Index: An Alternative Indicator for Measuring the Impacts of Meteorological Factors on Diarrhoea in the Climate Change Era: A Time Series Study in Dhaka, Bangladesh
Source: Int J Environ Res Public Health. 2024 Nov 7;21(11):1481. doi: 10.3390/ijerph21111481 (PMC11593466; doi:10.3390/ijerph21111481)
Supplement: Supplementary file 1 [file ijerph-21-01481-s001.zip › ijerph-3184339-supplementary/S1 File.pdf]

S1 File: Description of the health data

Health outcome data for this study was obtained from the icddr,b Dhaka Hospital. The hospital served an urban population of approximately 3.5 million in 1981, 6.6 million in 1990 and 14.6 million in 2010. The Dhaka Hospital treated approximately 110,000 (Range: 91,375−141,400) patients annually between 1991 and 2010. The hospital is located within the city limits and nearby the largest land transport depot in Dhaka, which make it geographically accessible to all city residents. However, healthcare seeking data show that the Dhaka Hospital's services are accessed predominantly by the disadvantaged group and surrounding slum dwellers.

Between 1970 and 1980, the number of patients seen at the hospital increased 10-fold from about 10 000 to about 100 000 patients a year, and it became difficult to collect useful information on all patients due to logistical constraints. As a result, the Diarrheal Disease Surveillance System (DDSS) was established at the Dhaka Hospital in 1979 to study the epidemiological, clinical, and laboratory characteristics of a sample of the patients who come to the hospital for care each year. The DDSS was approved by both the Research Review Committee and the Ethical Review Committee of icddr,b, and informed verbal consent was provided by the patients or by parents/attending caregivers for minors.

A systematic sub-sample of patients attending the Dhaka Hospital were enrolled into the surveillance program. During 1979 and 1995, every 25th patient (4% of the sample) and during 1996-2010, every 50th patient (2% of the sample) was enrolled into the DDSS. Surveillance patients were seen by the regular hospital staff with emergency cases treated on a priority basis. All patients were first treated in an outpatient area; those requiring further care were admitted to an intravenous treatment center or a hospital ward. After initial examination and care by a nurse or doctor, a trained health assistant sought verbal informed consent from the surveillance patient or an adult guardian prior to interview. Using a structured questionnaire, the trained health assistant interviewed patients and/or their caregivers and collected and recorded relevant information on socioeconomic and demographic characteristics, housing and environmental conditions, feeding practices of young children, and use of drugs and fluid therapy at home from diarrheal patients presenting to the icddr,b

Dhaka Hospital prior to receiving their diagnosis. Information on clinical characteristics, anthropometric measurements, and treatments received at the facilities and outcomes of patients were also recorded. A doctor performed a physical examination, including assessment of the state of dehydration. Dehydration was graded as none, mild (< 5%), moderate (5–10%), and severe (>10%) according to clinical signs. To assess nutritional state, each child 10 years or younger was weighed and measured at discharge.

All patients enrolled into the DDSS had their stool tested for enteric pathogens, including *V. cholera*, Shigella, Salmonella, rotavirus, amoeba, and Giardia species using microscopy, culture and enzyme-linked immunosorbent assay (ELISA). This surveillance activity provided valuable information to hospital clinicians in their clinical decision-making processes and enabled icddr,b to detect the emergence of new pathogens and in early identification of outbreaks and their locations and for monitoring changes in patients' characteristics and antimicrobial susceptibility of bacterial pathogens. This data constituted an important database for conducting epidemiological studies, validation of results of clinical studies, developing new research ideas and study designs, improving patient-care strategies and introducing preventive programs. Approximately, *V. cholerae* was identified in 30%, rotavirus in 20% and Shigella in 5% of the samples tested annually [1].

While individual data for all patients enrolled into the DDSS surveillance are available in the icddr,b DDSS database, this data set is not freely accessible. Given the limited resources available for this thesis, individual level data could not be accessed. Instead, grouped data including the total number of patients in the age-groups <5 years, 5–14 years, 15+ years who were hospitalized for all-cause diarrheal diseases at the icddr,b's Dhaka Hospital from 1 January 1981 to 31 December 2010 and who were enrolled into the DDSS was accessed for this study.

Describing the primary outcome

The primary outcome of interest for analysis of the thesis objectives is the total number of all-cause diarrhea patients hospitalized per day in the icddr,b Dhaka Hospital. However, this information was not recorded and hence not available in the Diarrheal Disease Surveillance System (DDSS) platform for the study period (1981–2010). The

DDSS platform only recorded the information of the all-cause diarrhea patients who were enrolled into the icddr,b hospital surveillance system. Furthermore, there was a change in the surveillance frequency between 1981–1995 and 1996–2010. During 1981–1995, the DDSS enrolled every 25th patient whereas the DDSS enrolled every 50th patient between 1996–2010 into the surveillance system. Given this change in the frequency of patients enrolled into the DDSS from Dhaka between 1981–1995 and 1996–2010, the daily number of diarrhea patients enrolled between 1981–1995 was multiplied by 25 and the patients enrolled between 1996–2010 were multiplied by 50 to estimate the total number of all-cause diarrhea patients seeking hospital care from the icddr,b Dhaka Hospital. This strategy allowed standardization and accounted for the change in surveillance frequency. Such standardization also provided the estimated total number of diarrhea patients seeking care from the icddr,b Dhaka Hospital per day, which is the primary outcome of interest for this study. In the absence of reliable records of all patients admitted into the hospital per day, this strategy was deemed acceptable locally and has been employed by the hospital authority to report the total number of admitted diarrhea cases per day to the national passive surveillance program (Diarrhea Control Program in Bangladesh) since 1981.

A case of diarrhea who required hospital treatment was considered as severe and the analysis was focused on these cases because severe cases are of greatest clinical significance. It is likely that predominantly infectious gastroenteritis (IG) cases were included in this study. However, a limited number of people who had chronic or persistent diarrhea at their first presentation and people with irritable bowel disease (IBD) who presented with similar symptoms were also likely included. Since, ambient temperature including heat wave affect both IG and IBD [2], and because it was logistically impossible to test all stool samples for all possible pathogens, a syndromic approach was regarded appropriate for this study.

References

1. Haque, F. Impacts of Temperature, Diurnal Temperature Range, Heat Index and Heat Wave on Diarrhoeal Diseases in Dhaka, Bangladesh in the Context of Climate Change: A Time-Series Analysis. Ph.D. Thesis, University College London (UCL), London, UK, 2022.

2. Haque, F.; Lampe, F.C.; Hajat, S.; Stavrianaki, K.; Hasan, S.M.T.; Faruque, A.S.G.; Ahmed, T.; Jubayer, S.; Kelman, I. Is heat wave a predictor of diarrhoea in Dhaka, Bangladesh? A time-series analysis in a South Asian tropical monsoon climate. PLOS Glob. Public Health 2024, 4, e0003629. https://doi.org/10.1371/journal.pgph.0003629. PMID: 39226251; PMCID: PMC11371214.
