# Supplementary material for: Heat Index: An Alternative Indicator for Measuring the Impacts of Meteorological Factors on Diarrhoea in the Climate Change Era: A Time Series Study in Dhaka, Bangladesh
Source: Int J Environ Res Public Health. 2024 Nov 7;21(11):1481. doi: 10.3390/ijerph21111481 (PMC11593466; doi:10.3390/ijerph21111481)
Supplement: Supplementary file 1 [file ijerph-21-01481-s001.zip › ijerph-3184339-supplementary/S2 File.pdf]

S2 File. Conceptual Framework

Table 1. Conceptual framework for considering factors as potential confounders/covariates/effect modifiers in the models investigating the relationship between heat index (explanatory/independent variable) and diarrhoea hospitalisation (response/dependent variable)

Factor	Level	Vary on a daily basis	Effect on exposure	Effect on outcome	Confounder/ covariate/ effect modifier
Rainfall	Population	Yes	Yes	Heavy rainfall increases the occurrence of diarrhoeal disease by increasing contamination of drinking water. Heavy rainfall increases host susceptibility to infection by causing malnutrition through crop/livestock destruction and reduced agricultural yield. Excess/scanty rainfall can cause floods/droughts and lead to microbial contamination of drinking water causing diarrhoea [1-4].	Confounder
Seasonality/Long-term trend	Population	Yes	Possible (seasonal scale)	Diarrhoea is seasonal. Cholera has a dual peak in the year [1, 3-5].	Confounder/covariate
Day of the week vs weekend	Population	Yes	May be	Access to healthcare and diarrhoea hospitalisation rates may vary between weekdays and weekends [1-4, 6].	Covariate
Age	Individual	No	None	Young age under 5 and adults over 70 years are more susceptible to diarrhoea mortality [3, 4, 7].	Effect modifier
Sex	Individual	No	None	Males have higher incidence than girls. However, males are likely to access healthcare more than females. Women may be more vulnerable due to caregiver responsibility and low access to healthcare [3, 4, 7].	Effect modifier
Population changes	Population	No	None	High population density is associated with more diarrhoea [3, 4, 7].	Effect modifier
Access to improved water supply	Population	No	None	Poor access to improved drinking water supply leads to excess diarrhoea [3, 4, 7].	Effect modifier
Sanitation coverage	Population	No	None	Poor sanitation coverage leads to excess diarrhoea [3, 4, 7].	Effect modifier

Investigating confounders/covariates

In statistics, a confounder (also known as a confounding variable, confounding factor, or lurking variable) is a variable that influences both the dependent variable and independent variable, causing a spurious association. Unlike normal regression analysis, in a time series analysis, the main unit of analysis is the day and not the individual person. This is an important point when considering what the potential confounders might be in the analysis [8]. In general epidemiology, common confounders

include age, gender and literacy level but these confounders do not apply to the time series regression analysis because at the population level, the distribution of these factors are unlikely to change on a day-to-day basis and cannot be associated with fluctuations in independent variables i.e. the environmental exposures such as heat index [8, 9]. Potential time-varying confounders such as cumulative rainfall and atmospheric pressure, which can change on a daily basis and may affect both the outcome and exposure of interest, was incorporated into the model to control for confounders. The procedure was repeated with the different meteorological variables. Only those associations for which meaningful correlations were found, were included in the final model allowing for non-linearity where expected [8]. We also incorporated covariates in the model. A covariate was a variable that was related to the dependent variable but was not the main focus of the study. These were included in the analysis to account for their potential influence on the dependent variable. Covariates were used to increase the precision and accuracy of estimates by reducing the error variance.

Investigating effect modification

In randomised controlled trials and regression models using individual data, modification of the effect of association by a third variable (i.e. effect modifier) can be examined using various approaches. Typically, factors of interest are tested and tried in models to assess whether these explain the variability in the outcome. Only if explanatory of the outcome of interest, a factor is tested if it affects the relationship of interest. The usual statistical approach for evaluating potential effect modifiers is a test for statistical interaction [6]. Frequently, interaction terms are included into the model and if found statistically significant (P-value <0.05), there is evidence that a synergy effect exists between these variables. However, in time series regression models using grouped data, such statistical tests of interactions cannot be applied [8, 9]. As a result, potential effect modifiers were identified a priori through review of the existing literature. Finally, sub-group analysis was conducted, and stratum-specific measures of association reported to highlight effect modification. Table 1S provides the conceptual framework for considering factors as potential confounders/covariates/effect modifiers.

References

1. Hashizume, M., et al., *Association between climate variability and hospital visits for non-cholera diarrhoea in Bangladesh: effects and vulnerable groups.* Int J Epidemiol, 2007. **36**(5): p. 1030-7.
2. Hashizume, M., et al., *Rotavirus infections and climate variability in Dhaka, Bangladesh: a time-series analysis.* Epidemiol Infect, 2008. **136**(9): p. 1281-9.
3. Levy, K., S.M. Smith, and E.J. Carlton, *Climate Change Impacts on Waterborne Diseases: Moving Toward Designing Interventions.* Curr Environ Health Rep, 2018. **5**(2): p. 272-282.
4. Levy, K., et al., *Untangling the Impacts of Climate Change on Waterborne Diseases: a Systematic Review of Relationships between Diarrheal Diseases and Temperature, Rainfall, Flooding, and Drought.* Environ Sci Technol, 2016. **50**(10): p. 4905-22.

5. Huq, A., et al., *Critical factors influencing the occurrence of Vibrio cholerae in the environment of Bangladesh.* Appl Environ Microbiol, 2005. **71**(8): p. 4645-54.
6. Imai, C., et al., *Time series regression model for infectious disease and weather.* Environ Res, 2015. **142**: p. 319-27.
7. Haque, F., *Impacts of temperature, diurnal temperature range, heat index and heat wave on diarrhoeal diseases in Dhaka, Bangladesh in the context of climate change: A time-series analysis*, in *Institute for Global Health (IGH)*. 2022, University College London (UCL): London
8. Bhaskaran, K., et al., *Time series regression studies in environmental epidemiology.* Int J Epidemiol, 2013. **42**(4): p. 1187-95.
9. Shumway, R.H. and D.S. Stoffer, eds. *Time Series Analysis and Its Applications* Fourth ed. Springer Texts in Statistics 2017, Springer International Publishing AG: Switzerland
