# Supplementary material for: Heat Index: An Alternative Indicator for Measuring the Impacts of Meteorological Factors on Diarrhoea in the Climate Change Era: A Time Series Study in Dhaka, Bangladesh
Source: Int J Environ Res Public Health. 2024 Nov 7;21(11):1481. doi: 10.3390/ijerph21111481 (PMC11593466; doi:10.3390/ijerph21111481)
Supplement: Supplementary file 1 [file ijerph-21-01481-s001.zip › ijerph-3184339-supplementary/S3 File.pdf]

S3 File: Sensitivity analyses and model diagnostics

Sensitivity analysis was conducted by varying the amount of control for long-term trend and seasonality using established approaches [1-4]. Initially, the analyses were repeated using 1-14 degrees of freedom per year to control for long-term trend and seasonality in order to check the robustness of the results.

$$\log[E(Y_t)] = \beta_0 + \beta_1 HI_{1t} + \beta_2 Rain_{2t} + \beta_3 ATPress_{3t} + \gamma_1 dow_{1t} + \gamma_2 Season_{2t} + \vartheta_1(LS\ Time,\ 1\text{-}14\ D.F.)_{1t} \quad (1\text{-}1)$$

In addition, a natural cubic spline with 3-7 D.F. was incorporated into the model to control for long-term trend instead of fitting linear splines to time.

For the sensitivity analysis, the model took the following form:

$$Y_t \sim Negative\ Binomial\ (\mu t,\ \theta)$$

$$\log[E(Y_t)] = \beta_0 + \beta_1 HI_{1t} + \beta_2 Rain_{2t} + \beta_3 ATPress_{3t} + \gamma_1 dow_{1t} + \gamma_2 Season_{2t} + \vartheta_1(NS\ Time,\ 3\text{-}7\ D.F.)_{1t} \quad (1\text{-}2)$$

To explore to what extent the changes in the sample size affected the results, the models were re-run using the actual number of diarrheal disease patients enrolled into the surveillance system (DDSS) instead of the total estimated number of patients with diarrheal diseases in the Dhaka Hospital during the study period.

$$\log[E(DDSS_t)] = \beta_0 + \beta_1 HI_{1t} + \beta_2 Rain_{2t} + \beta_3 ATPress_{3t} + \gamma_1 dow_{1t} + \gamma_2 Season_{2t} + \vartheta_1(LS\ Time,\ 1\text{-}14\ D.F.)_{1t} \quad (1\text{-}3)$$

In addition, we used a generalised linear model to study the effects of interaction of temperature and relative humidity on diarrhoea hospitalization. The model took the following form:

$$log[E(Y_t)] = \beta_0 + \beta_1 Temp_{1t} + \beta_2 Hum_{2t} + \beta_3 Rain_{3t} + \beta_4 ATPress_{4t} + \delta_1 (Temp_{1t} \times Hum_{2t}) + \gamma_1 dow_{1t} + \gamma_2 Season_{2t} + \vartheta_1 (LS\ Time,\ 8\ D.F.)_{1t} \quad \ldots\ldots (1\text{-}4)$$

Findings

The results of the sensitivity analysis are shown in S1 Table. All results are comparable to the original analysis. The models with the lowest BIC values were considered as the best models.

S1 Table. Percentage increase of diarrhea hospitalisation associated with one unit increase in heat index (HI).

**HI**	**Percentage increase (95% CI)**							
	**All ages**		**Under-5 children**		**Males**		**Females**	
D.F.	Using Equation 1-1							
	IRR (95% CI)	*p*-value	IRR (95% CI)	p-value	IRR (95% CI)	p-value	IRR (95% CI)	p-value
1	0.59 (0.52 – 0.66)	<0.001	1.29 (1.03 – 1.59)	<0.001	1.06 (0.78 – 1.35)	0.001	1.02 (0.63 – 1.28)	0.003
2	0.62 (0.55 – 0.70)	<0.001	1.29 (1.04 – 1.51)	<0.001	1.06 (0.74 – 1.43)	0.001	1.02 (0.63 – 1.27)	0.003
3	0.62 (0.55 – 0.69)	<0.001	1.31 (1.09 – 1.51)	<0.001	1.04 (0.74 – 1.44)	0.001	1.01 (0.63 – 1.27)	0.003
4	0.66 (0.59 – 0.74)	<0.001	1.30 (1.05 – 1.59)	<0.001	1.05 (0.88 – 1.33)	0.001	1.01 (0.64 – 1.28)	0.003
5	0.72 (0.65 – 0.79)	<0.001	1.32 (1.11 – 1.53)	<0.001	1.07 (0.89 – 1.34)	0.001	1.01 (0.66 – 1.26)	0.003
6	0.78 (0.70 – 0.85)	<0.001	1.32 (1.12 – 1.52)	<0.001	1.07 (0.91 – 1.39)	0.001	1.00 (0.69 – 1.27)	0.003
7	0.84 (0.77 – 0.92)	<0.001	1.32 (1.13 – 1.52)	<0.001	1.09 (0.84 – 1.37)	0.001	1.00 (0.70 – 1.27)	0.003
8*	0.84 (0.76 – 0.91)	<0.001	1.32 (1.13 – 1.51)	<0.001	1.10 (0.92 – 1.29)	<0.001	1.00 (0.73 – 1.27)	0.002
9	0.85 (0.78 – 0.92)	<0.001	1.32 (1.09 – 1.62)	<0.001	1.08 (0.91 – 1.31)	0.001	1.00 (0.79 – 1.32)	0.002
10	0.88 (0.80 – 0.95)	<0.001	1.32 (1.05 – 1.62)	<0.001	1.07 (0.91 – 1.38)	0.001	1.03 (0.81 – 1.35)	0.001
11	0.87 (0.80 – 0.95)	<0.001	1.32 (1.06 – 1.61)	<0.001	1.06 (0.88 – 1.34)	0.001	1.02 (0.80 – 1.37)	0.004
12	0.87 (0.80 – 0.94)	<0.001	1.31 (1.06 – 1.60)	<0.001	1.05 (0.86 – 1.37)	0.001	1.02 (0.81 – 1.37)	0.002
13	0.87 (0.79 – 0.94)	<0.001	1.31 (1.07 – 1.67)	<0.001	1.05 (0.93 – 1.34)	0.001	1.04 (0.79 – 1.27)	0.002
14	0.88 (0.81 – 0.96)	<0.001	1.31 (1.06 – 1.65)	<0.001	1.06 (0.91 – 1.34)	0.001	1.04 (0.79 – 1.29)	0.002
D.F.	Using Equation 1-2							
3	0.46 (0.39 – 0.53)	<0.001	1.21 (1.16 – 1.63)	<0.001	1.03 (0.73 – 1.33)	0.001	1.01 (0.69 – 1.27)	0.003
4	0.48 (0.41 – 0.55)	<0.001	1.22 (1.15 – 1.65)	<0.001	1.03 (0.75 – 1.38)	0.001	1.00 (0.69 – 1.29)	0.003
5	0.54 (0.47 – 0.61)	<0.001	1.21 (1.15 – 1.61)	<0.001	1.04 (0.79 – 1.35)	0.001	1.01 (0.71 – 1.25)	0.003
6*	0.54 (0.47 – 0.62)	<0.001	1.22 (1.22 – 1.59)	<0.001	1.06 (0.81 – 1.35)	0.001	1.00 (0.70 – 1.27)	0.003
7	0.54 (0.47 – 0.61)	<0.001	1.22 (1.16 – 1.61)	<0.001	1.07 (0.80 – 1.36)	0.001	1.01 (0.73 – 1.29)	0.003
D.F.	Using Equation 1-3							
8*	0.72 (0.46 – 0.99)	<0.001	1.29 (1.02 – 1.59)	<0.001	1.08 (0.62 – 1.36)	0.002	1.00 (0.77 – 1.32)	0.005
D.F.	Using Equation 1-4							
8*	0.64 (0.57 – 0.94)	<0.001	1.26 (1.05 – 1.61)	<0.001	1.09 (0.53 – 1.41)	0.002	1.00 (0.69 – 1.39)	0.004

*Represents the model with the lowest BIC values.

S1 Fig. Partial autocorrelation function plot of deviance residuals of the heat index regression models using Equation 1-1 with 8 D.F. for all ages (upper left), under-5 children (upper right), males (lower left) and females (lower right) adjusted for autocorrelation

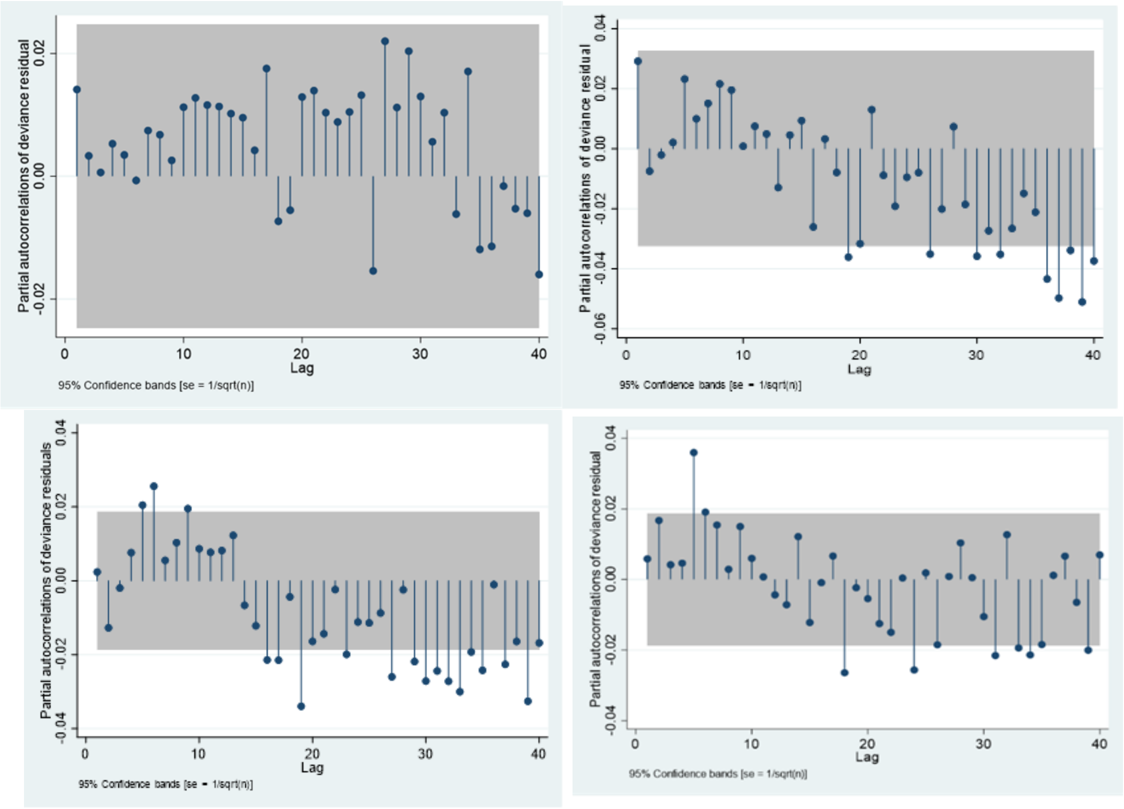

S2 Fig. Partial autocorrelation function plot of deviance residuals of the extremely high heat index regression models using Equation 1-1 with 8 D.F. for all ages (upper left), under-5 children (upper right), males (lower left) and females (lower right) adjusted for autocorrelation

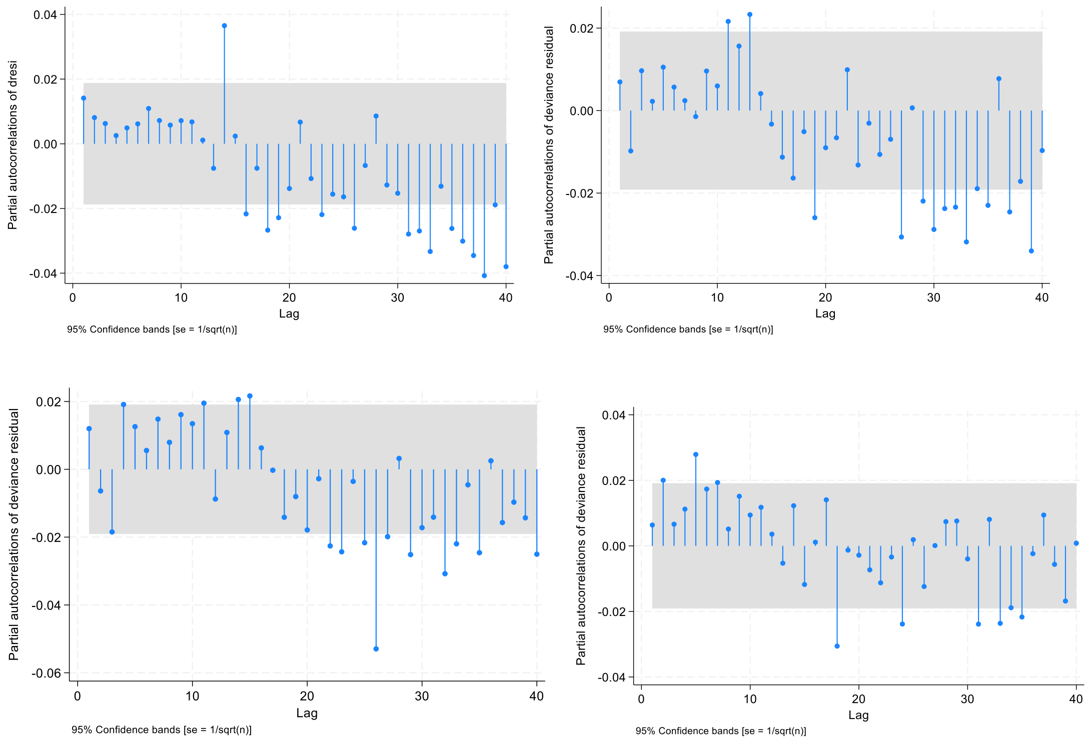

S3 Fig. Partial autocorrelation function plot of deviance residuals of the very high heat index regression models using Equation 1-1 with 8 D.F. for all ages (upper left), under-5 children (upper right), males (lower left) and females (lower right) adjusted for autocorrelation

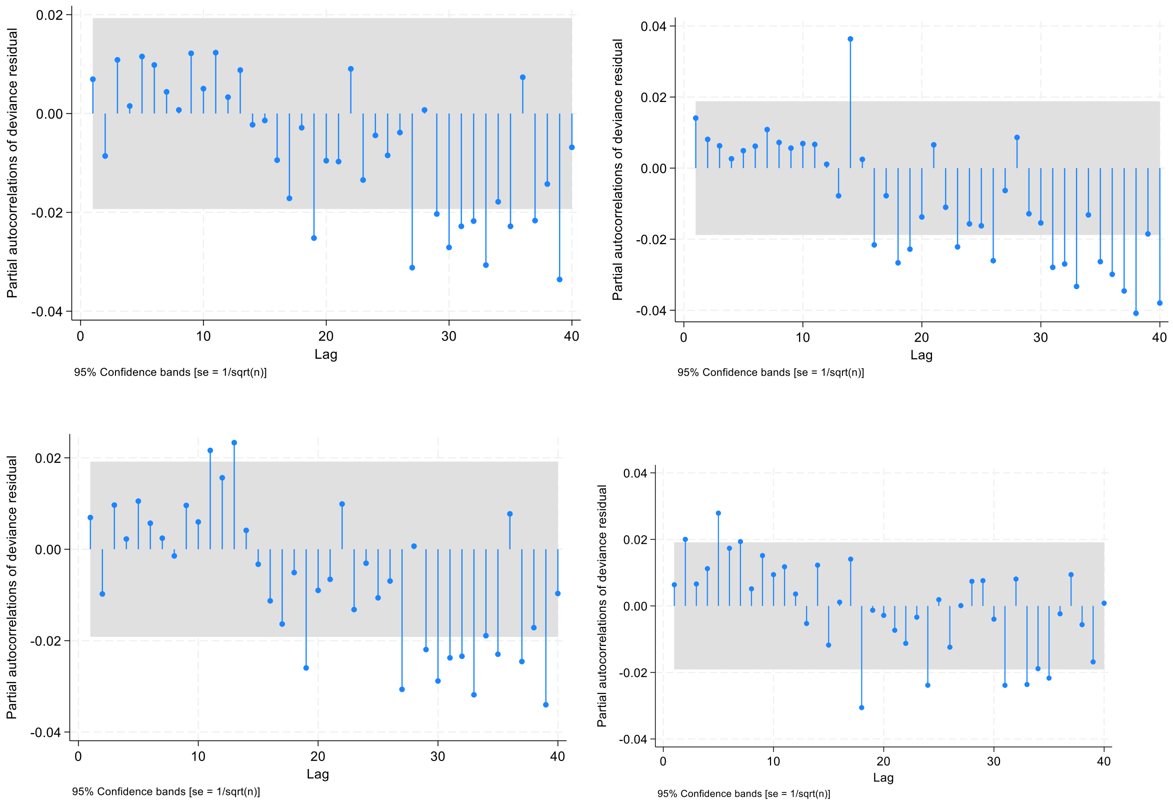

S4 Fig. Partial autocorrelation function plot of deviance residuals of the high heat index regression models using Equation 1-1 with 8 D.F. for all ages (upper left), under-5 children (upper right), males (lower left) and females (lower right) adjusted for autocorrelation

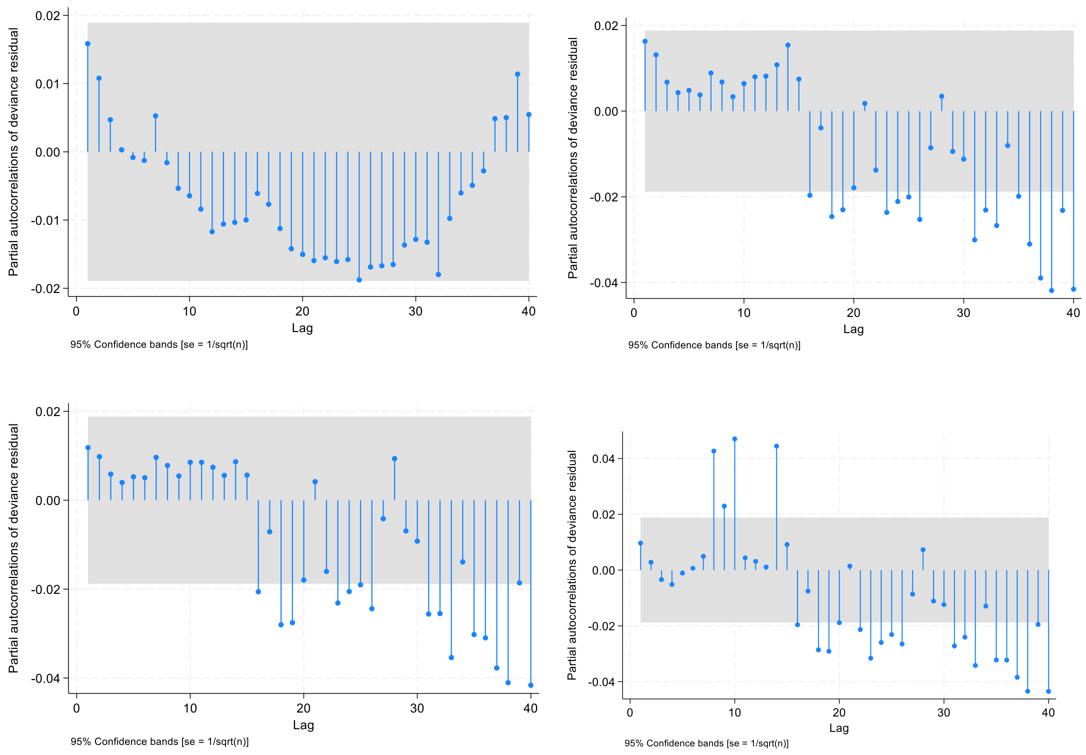

References

1. Hardin, J.W.; Hilbe, J.M. (Eds.) Generalized Linear Models and Extensions, 2nd ed.; Stata Press: College Station, TX, USA, 2007.

2. Chandler, R.E.; Scott, E.M. (Eds.) Statistical Methods for Trend Detection and Analysis in the Environmental Sciences, 1st ed.; John Wiley & Sons, Ltd.: Chichester, UK, 2011.

3. Imai, C.; Armstrong, B.; Chalabi, Z.; Mangtani, P.; Hashizume, M. Time series regression model for infectious disease and weather. Environ. Res. 2015, 142, 319–327. https://doi.org/10.1016/j.envres.2015.06.040. PMID: 26188633.

4. Bhaskaran, K.; Gasparrini, A.; Hajat, S.; Smeeth, L.; Armstrong, B. Time series regression studies in environmental epide-miology. Int. J. Epidemiol. 2013, 42, 1187–1195. https://doi.org/10.1093/ije/dyt092. PMID: 23760528; PMCID: PMC3780998.
